# Postoperative (chemo) radiation in patients with squamous cell cancers of the head and neck – clinical results from the cohort of the clinical cooperation group “Personalized Radiotherapy in Head and Neck Cancer”

**DOI:** 10.1186/s13014-018-1067-1

**Published:** 2018-07-03

**Authors:** Cornelius Maihoefer, Lars Schüttrumpf, Corinna Macht, Ulrike Pflugradt, Julia Hess, Ludmila Schneider, Christine Woischke, Axel Walch, Philipp Baumeister, Thomas Kirchner, Horst Zitzelsberger, Claus Belka, Ute Ganswindt

**Affiliations:** 1 Clinical Cooperation Group ‘Personalized Radiotherapy in Head and Neck Cancer’, Helmholtz Zentrum München, German Research Center for Environmental Health GmbH, Ingolstädter Landstraße 1, 85764 Neuherberg, Germany; 2 Department of Radiation Oncology, University Hospital, LMU Munich, Marchioninistr. 15, 81377 Munich, Germany; 3 Gemeinschaftspraxis für Strahlentherapie und Radioonkolgie am Klinikum Schwabing, Kölner Platz 1, 80804 Munich, Germany; 4 Research Unit Radiation Cytogenetics, Helmholtz Zentrum München, German Research Center for Environmental Health GmbH, Ingolstädter Landstraße 1, Neuherberg, 85764 Germany; 5 Department of Otorhinolaryngology, Head and Neck Surgery, University Hospital, LMU Munich, Marchioninistr. 15, 81377 Munich, Germany; 6 Institute of Pathology, Faculty of Medicine, LMU Munich, Marchioninistr. 27, 81377 Munich, Germany; 7 Research Unit Analytical Pathology, Helmholtz Zentrum München, German Research Center for Environmental Health GmbH, Ingolstädter Landstraße 1, 85764 Neuherberg, Germany; 8 Department of Radiation Oncology, University of Innsbruck, Anichstraße 35, 6020 Innsbruck, Austria

**Keywords:** Head and neck cancer, Squamous cell carcinoma, Postoperative, Adjuvant, Chemoradiation, Radiation therapy, Radiotherapy, HPV, HNSCC

## Abstract

**Background:**

Postoperative (chemo) radiation improves tumor control and survival in high-risk patients with head and neck squamous cell carcinoma based on established risk factors. The clinical cooperation group “Personalized Radiotherapy in Head and Neck Cancer” focuses on the identification and validation of new biomarkers, which are aimed at eventually stratifying and personalizing the therapy concept. Hence, we reviewed all patients with head and neck squamous cell carcinoma of the oral cavity, oropharynx, hypopharynx, or larynx, treated with postoperative (chemo) radiation from 06/2008 until 06/2015 at the Department of Radiation Oncology in the University Hospital, LMU Munich. Here we report the clinical results of the cohort, laying the foundation for further research within the framework of a clinical cooperation group.

**Methods:**

Patient data were retrospectively (until 2013) and prospectively (from 2013) collected and analyzed for outcome and treatment failures with regard to previously described and established risk factors.

**Results:**

We identified 302 patients (median follow-up 45 months, average age 60.7 years), having received postoperative (chemo)radiation (median 64 Gy). Chemotherapy was added in 58% of cases, mostly Cisplatin/5- Fluorouracil in concordance with the ARO 96–3 study. The 3-year overall survival, local, locoregional and distant failure estimates were 70.5, 9.7, 12.2 and 13.5%, respectively. Human papillomavirus-associated oropharyngeal cancer was associated with a significant improved overall survival, locoregional, distant and overall tumor control rates in multivariate analysis. Additionally, in multivariate analysis, for local failure, resection status and perineural invasion, for locoregional and distant failure extracapsular extension and for overall survival the presence of nodal disease were significant adverse factors. Moreover, 138 patients have been treated in concordance with the ARO 96–3 protocol, corroborating the results of this study.

**Conclusions:**

Our cohort represents a large unselected cohort of patients with head and neck squamous cell carcinoma treated with postoperative (chemo)radiation. Tumor control rates and survival rates are consistent with the results of previously reported data.

## Background

Postoperative (chemo)radiation is the standard treatment for patients with head and neck squamous cell carcinoma (HNSCC) presenting with established risk factors such as large primary tumors, positive nodal involvement, and close or incomplete resection margins after surgery [1–7]. Since the joint analysis by Cooper and Bernier, close or incomplete resection margins or lymph nodes with extracapsular spread are established risk factors for the indication of additional chemotherapy [8, 9]. However, the prognosis of patients with HNSCC is still to be improved [10, 11]. At the same time, Human papillomavirus (HPV)-associated oropharyngeal carcinoma (HPVOPC) have a much better outcome than HPV-negative HNSCC [12–15].

Due to rigid inclusion and exclusion criteria, most studies do not represent the usual “everyday” patient. Here we describe an unselected cohort of patients that have been treated with postoperative (chemo)radiation in our department. This cohort (LMU-KKG) lays the foundation for ongoing and future research such as the establishment of new biomarkers and the personalization of head and neck oncology in the context of the multidisciplinary translational Clinical Cooperation Group (CCG; german: KKG) “Personalized Radiotherapy in Head and Neck Cancer”.

## Methods

We analyzed 302 patients with squamous cell carcinoma of the oral cavity, oropharynx, hypopharynx and larynx who have been treated with postoperative radiation therapy in our clinic (Department of Radiation Oncology, University Hospital, LMU Munich) between 06/2008 and 06/2015 retrospectively (until 2013) and prospectively (from 2013). From 2013 onwards, the data acquisition was conducted prospectively within the framework of the clinical cooperation group “Personalized Radiotherapy in Head and Neck Cancer”. Patients aged at least 18 years with the aforementioned tumor sites and histology were included. Basically, all patients with HNSCC were permitted in the prospective collection cohort, but only those with surgery followed by adjuvant (chemo)radiation were included in this analysis.

Patients with risk factors such as large primary tumors (pT3/pT4), positive nodal involvement (≥pN1), close (< 5 mm), incomplete resection margins or in some cases previously not irradiated recurrent tumors were treated with postoperative radiation therapy. The advised dose was usually 64–66 Gy to the former tumor bed, 50–54 Gy to the elective nodal levels and 56–60 Gy to involved nodal levels; both 3D- and IMRT-technique (intensity-modulated radiotherapy) have been used. In cases of close or incomplete resection margins or lymph nodes with extracapsular extension (ECE) patients underwent additional chemotherapy, consisting of Cisplatin/5-Fluorouracil (CDDP/5-FU) in concordance with the ARO 96–3 Study (CDDP (20 mg/m^2^ on day 1–5, 29–33) and 5-FU (600 mg/m^2^ on day 1–5, 29–33) [9]. The reasons for selecting this regimen were positive treatment experiences during the participation in the study and the promising results presented at the ASCO-Meeting in 2009. However, in 2016 this regimen was discontinued in favor of CDDP mono, as no published solid long-term data further supported the CDDP/5-FU approach. Other chemotherapeutic regimens (such as CDDP 40 mg/m^2^ weekly, Mitomycin C (MMC) 10 mg/m^2^ d1,d29; 5-FU 600 mg/m^2^ d1–5, MMC 10 mg/m^2^ d5,d36 or Cetuximab 250 mg/m^2^ weekly with 400 mg/m^2^ loading dose) were used if a patient with clear indication for chemoradiation was not suitable for combined CCDP and 5-FU-based chemotherapy due to relevant comorbidities or advanced age (e.g. in case of decreased renal function 5-FU/MMC, in case of cardiac comorbidities CDDP only or in case of advanced age and reduced performance status switch to MMC or Cetuximab mono).

The clinic’s radiation therapy management system (Mosaiq® - Elekta, Sweden) and patient files recorded in a dedicated Microsoft® Access® Relational Database were used to collect patient data.

Tumor stage was assessed using the UICC 7th edition classification, for HPVOPC also UICC 8th edition stages were added. Resection margins were considered “close margin” when declared R0, but less than 5 mm by the local pathologist. Other risk factors were recurrent disease, lymphovascular invasion (LVI), venous tumor invasion (VTI), perineural invasion (PNI), extracapsular extension (ECE) and number of involved lymph nodes. Immunohistochemical (IHC) p16^INK4a^ staining results from our local pathology was used as a surrogate marker for HPV-infection, if available (162 patients). Additionally, 124 of the remaining HNSCC patients were analyzed for HPV p16 within the framework of the KKG. IHC p16^INK4a^ staining was performed using the CINtec TM Histology Kit (Roche mtm laboratories AG, Heidelberg, Germany) on a Ventana Benchmark LT automated immunostainer (Ventana Medical Systems, Tucson AZ, USA) according to the protocol. Tumor specimens with strong and diffuse nuclear and cytoplasmic staining in more than 70% of tumor cells were considered as p16-positive.

Follow-up data has been gathered in the joint survivorship clinic of the Otorhinolaryngological and the Radiation Oncology Department of the LMU, but also from follow-up visits in our clinic, medical records from external care givers or by telephone (for assessing the survival only).

Follow-up has been calculated from the first day of radiation therapy with the reverse Kaplan-Meier estimate [16]. All other endpoints such as survival or time to recurrence have been calculated from the first day of the radiation treatment. The events of the survival endpoints were defined as following: overall survival (OS) – death, disease-free survival (DFS) – death or any recurrence, disease-specific survival (DSS) – only death related to recurring HNSCC. *P*-values were determined using log-rank testing for comparison between groups. Univariate and multivariate analyses were conducted using Cox proportional hazard regression models. If more than one factor was significant in univariate Cox regression analysis, multivariate Cox regression analysis with backward elimination (likelihood ratio) was used for determining the influence of multiple covariates. If possible by the number of events, factors with *p* < 0.1 were also included in multivariate analysis. Statistical analyses were performed with SPSS V24 (IBM, Chicago, IL) and R (Version 3.3.1). *P*-values of < 0.05 were considered statistically significant. Median estimates and Hazard ratios (HR) with 95% confidence intervals (CI) were determined. Ethics approval for data gathering and the assessment of tumor probes were granted by the local ethics committee (No. 448–13, 459–13, 17–116).

## Results

### Patient and treatment characteristics

A total of 302 patients with HNSCC of the oral cavity, oropharynx, hypopharynx and larynx were treated with adjuvant (chemo)radiation therapy in our department between 06/2008 and 06/2015. Patient, tumor and treatment characteristics are shown in Table 1. The patients’ average age was 60.7 (range 20–87 yr., IQR 54–68 yr) at time of diagnosis. The median follow-up estimate was 45.0 months (95% CI 41.2–48.8 months, reverse Kaplan-Meier). 97% of patients completed radiation therapy and received at least 60 Gy to the tumor bed. 58% of patients (*n* = 176) received concurrent systemic therapy.

**Table 1:** Patient and treatment characteristics for all patients (left panel) recurrent patients (middle-left panel) and the ARO-analogue subgroup (middle-right panel) and patients with HPV-p16 positive oropharyngeal carcinoma (right panel)

Factors	All patients *n* = 302		Recurrent *n* = 24		ARO-analogue *n* = 138		HPVOPC *n* = 60	
	Number	Percent	Number	Percent	Number	Percent	Number	Percent
Primary Tumor								
Oropharynx	149	49.3	4	16.7	76	55.1	60	100.0
Oral cavity	63	20.9	6	25.0	20	14.5	0	0.0
Hypopharynx	39	12.9	3	12.5	24	17.4	0	0.0
Larynx	51	16.9	11	45.8	18	13.0	0	0.0
Sex								
male	226	74.8	18	75.0	108	78.3	40	66.7
female	76	25.2	6	25.0	30	21.7	20	33.3
Age								
< 45	11	3.6	0	0	5	3.6	5	8.3
45–54	66	21.9	5	20.8	36	26.1	6	10.0
55–64	114	37.7	7	29.2	63	45.7	24	40.0
65–74	88	29.1	9	37.5	32	23.2	19	31.7
> 75	23	7.6	3	12.5	2	1.4	6	10.0
UICC Stage 7th edition (^a^ 8th edition for HPVOPC)								
I	7	2.3	4	16.7	0	0.0	1 (42^a^)	1.7 (70.0^a^)
II	30	9.9	0	0	5	3.6	4 (16^a^)	6.7 (26.7^a^)
III	78	25.8	7	29.2	23	16.7	11 (2^a^)	18.3 (3.3^a^)
IV	187	61.9	13	54.2	110	79.7	44 (0^a^)	73.3 (0.0^a^)
Diagnosis class								
First diagnosis	272	90.1	0	0	125	90.6	59	98.3
Recurrence	24	7.9	24	100.0	11	8.0	1	1.7
Second primary	6	2.0	0	0	2	1.4	0	0
T-Stage								
T0	2	0.7	2	8.3	2	1.4	0	0.0
T1	56	18.5	6	25.0	28	20.3	12	20.0
T2	121	40.1	1	4.2	44	31.9	36	60.0
T3	74	24.5	6	25.0	40	29.0	10	16.7
T4	49	16.2	9	37.5	24	17.4	2	3.3
N-Stage								
N0	89	29.5	12	50.0	19	13.8	8	13.3
N1	58	19.2	6	25.0	23	16.7	9	15
N2a	23	7.6	0	0	15	10.9	10	16.7
N2b	85	28.1	5	20.8	47	34.1	25	41.7
N2c	42	13.9	1	4.2	30	21.7	8	13.3
N3	5	1.7	0	0	4	2.9	0	0
M-Stage								
M0	302	100	24	100	138	100	60	100
M1	0	0	0	0	0	0	0	0
Resection status (R-Status)								
R0	150	51.0	7	33.3	47	35.1	26	44.1
R0-Close margin	75	25.5	7	33.3	43	32.1	12	20.3
R1	69	23.5	7	33.3	44	32.8	21	35.6
N/A	8		3		4		1	
Extracapsular extension (ECE)								
no (N0)	89	29.8	12	52.5	19	13.9	8	13.3
no (N+)	132	44.1	6	26.1	59	43.1	34	56.7
yes	78	26.1	5	21.7	59	43.1	18	30.0
N/A	3		1		1		0	
Perineural invasion (PNI)								
no (Pn0)	198	83.5	8	88.9	86	82.7	41	87.2
yes (Pn1)	39	16.5	1	11.1	18	17.3	6	12.8
N/A	65		15		34		13	
Lymphatic Invasion (LI)								
no (L0)	201	73.4	11	78.6	82	66.1	40	69.0
yes (L1)	73	26.6	3	21.4	42	33.9	18	31.0
N/A	28		10		14		2	
Vascular Invasion (VI)								
no (V0)	266	97.1	12	100	119	96.0	55	96.5
yes (V1)	8	2.9	0	0	5	4.0	2	3.5
N/A	28		12		14		3	
Grading								
G1	9	3.0	2	8.7	2	1.5	0	0.0
G2	119	39.5	11	47.8	45	32.8	11	18.3
G3	173	57.5	10	43.4	90	65.7	49	81.7
N/A	1		1		1		0	
HPV p16								
negative	159	68.2	11	73.3	66	61.7	0	0.0
positive	74	31.8	4	26.7	41	38.8	60	100.0
HPVOPC	60	20.5	1	6.7	36	25.2	60	100.0
N/A	69		9		31		0	
RT dose in tumor bed								
< 60 Gy (not completed)	9	3	2	8.3	4	2.9	2	3.3
60–63.9 Gy	8	2.6	0	0	6	4.3	3	5.0
64 Gy	263	87.1	21	87.5	120	87	52	86.7
66 Gy	22	7.3	1	4.2	8	4.8	3	5.0
RT technique								
3d-conformal	245	81.1	21	87.5	116	84.1	41	68.3
IMRT	57	18.9	3	12.5	22	15.9	19	31.7
Concomitant chemotherapy								
no Chx	126	41.7	10	41.7	0	0.0	16	26.7
despite indicated	43	14.2	5	20.8	0	0.0	4	6.7
Med.	20		4		0	0.0	2	3.3
Pat. Refusal	23		1		0	0.0	2	3.3
Any Chemo	176	58.3	14	58.3	138	100.0	44	73.3
CDDP/5-FU	138	78.4	11	45.8	138	100.0	36	81.8
CDDP mono	9	5.1	1	4.2	0	0.0	3	6.8
Cetuximab	1	0.6	0	0	0	0.0	0	0.0
5-FU/MMC	8	4.5	1	4.2	0	0.0	3	6.8
MMC	20	11.4	1	4.2	0	0.0	2	4.5
Chemo completed	147	83.5	14	58.3	120	87.0	36	81.8
Chemo stopped	29	16.5	0		18	13.0	8	18.1
patient refused	8		0		6		2	
Worsening condition	7		0		3		3	
cytopenia	9		0		4		1	
reaction to chemo	5		0		5		2	
Death causes								
Tumor-related	42		3		21		1	
comorbidities	40		3		13		6	
therapy-associated	2		0		0		1	
Second primary	8		0		4		0	
other	5		0		3		0	

^a^The UICC 8th edition stage is shown in parenthesis (HPVOPC only)

### Tumor control rates and survival data for all patients

**Fig. 1:**
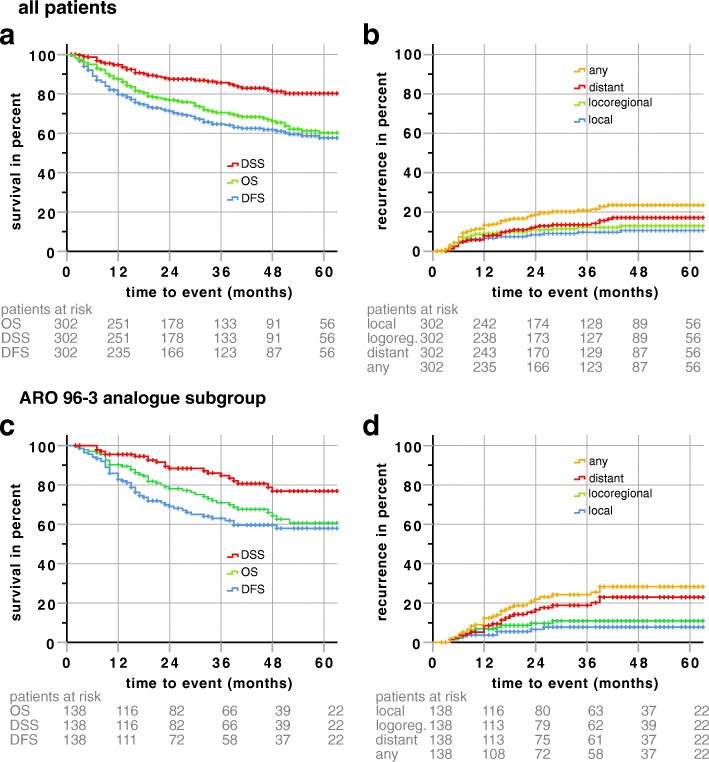
Kaplan-Meier plots **a** Overall survival (OS), disease-free survival (DFS) and disease-specific survival (DSS) of all patients **b** local, locoregional, distant and any failure rates of all patients. **c** overall survival (OS), disease-free survival (DFS) and disease-specific survival (DSS) of the ARO-analogue subgroup **d** local, locoregional, distant and any failure rates of the ARO-analogue subgroup. Follow-up time was clipped at 60 months. Patients at risk are displayed under the respective plots. Censors are represented by crosses. *P*-Values (Log Rank) are shown

For all patients 3 (5) year overall survival estimates were 70.5% (60.2%). The estimated disease-specific and disease-free survival rates were 85.7 and 64.7% after 3 years, respectively (Fig. 1a). The estimated 3-year failure rates were 9.7% for local, 12.2% for locoregional, 13.5% for distant and 20.8% for overall failures (Fig. 1b).

### Tumor control rates and survival data for patients treated for recurrent tumors

Patients with tumor recurrence after surgery have also been treated in the reported timeframe (*n* = 24; see Table 1). For patients treated due to recurrent disease 3(5)-year overall survival estimates were 74.6% (65.3%) in comparison to those not treated for recurrence with 69.6% (59.7%) (not significant; n.s.). The estimated 3-year disease-specific (disease-free) survival rates were 85.3% (67.4%) for the patients treated for recurrence and 85.0% (64.5%) for the patients treated for the first manifestation of the tumor (n.s.). The estimated 3-year failure rates were 15.8% for locoregional, 7.7% for distant and 22.8% for overall failures in the recurrent cohort and 11.9, 14.0 and 20.3% in the non-recurrent cohort (n.s.).

### Tumor control rates and survival data for the ARO 96–3 subgroup

One hundred thirty-eight patients were treated with CDDP/5-FU in concordance to the chemotherapy arm of the ARO 96–3 study, the patient’s characteristics are described in Table 1. The estimated median follow-up was 45 months (95% CI 40.5–49.5 month). 87% of patients received 2 cycles of chemotherapy; the remaining patients did not receive both cycles due to various reasons (refusal, worsening condition). The estimated 5-year OS, DFS and DSS were 60.6, 56.8 and 76.8%, respectively (Fig. 1c). The estimated 3-year local, locoregional and distant failure rates were 7.8, 10.9 and 18.9%, respectively (Fig. 1d). HPV-p16-status was associated with a significantly improved locoregional control, distant control, DFS, DSS and OS in the ARO-analogue group (data not shown).

### Risk factors

**Fig. 2:**
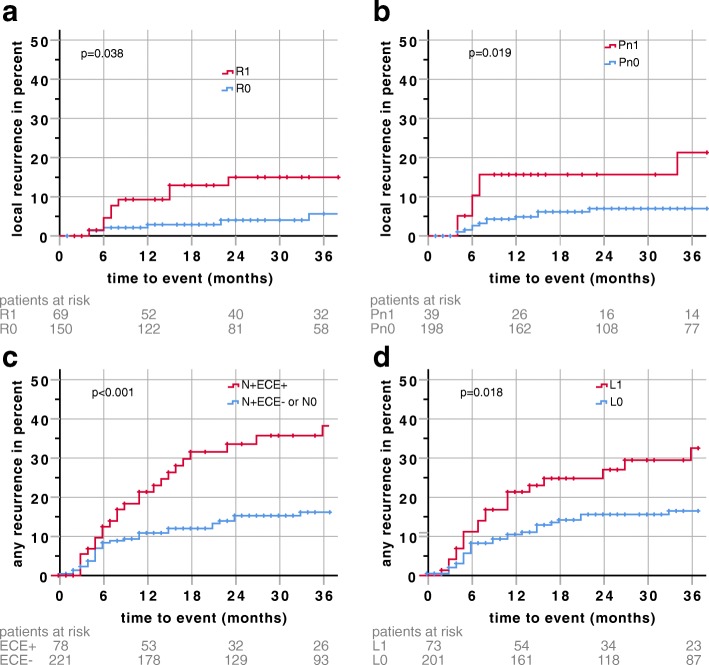
Exemplary Kaplan-Meier plots for risk factors that are significant for recurrence in multivariate analysis. **a** Local recurrence and resection status (R1 vs R0) **b** Local recurrence and perineural invasion (Pn1 vs Pn0) **c** any recurrence and extracapsular extension (ECE vs no ECE/N0) **d** any recurrence and lymphovascular invasion (L1 vs L0). *P*-values (log rank) of the Kaplan-Meier estimates are shown. Follow-up time was clipped at 36 months. Patients at risk are displayed under the respective plots. Censors are represented by crosses

Previously described risk factors, such as large primary tumors (pT3 or pT4) the presence of positive lymph nodes with and without extranodal disease (ECE), positive resection margins (R1), lymphangiosis (LVI), perineural invasion (PNI) and positive prognostic factors such as HPV-p16-positive oropharyngeal carcinoma (HPVOPC) were analyzed for correlation with local control, locoregional control, distant control, any control, overall survival (OS), disease-specific survival (DSS) and disease-free survival (DFS) in univariate and multivariate cox regression analysis (see Table 2, Fig. 2a-d).

**Table 2:** ᅟ

	Univariate			Multivariate		
	HR	95% CI	p-Value	HR	95% CI	p-Value
Local failure (LF)						
HPVOPC	0.128	0.017-0.945	0.044*^a^	0.146	0.019-1.115	0.064
≥T3	1.580	0.731-3.413	0.244	-	-	-
ECE vs N+ ^b^	2.849	1.014-8.007	0.047*^a^	2.262^d^	0.711-7.199	0.167
N+ vs N0 ^b^	0.350	0.129-0.946	0.039*^a^	0.555^d^	0.173-1.779	0.322
pos. Margins^c^	2.730	1.016-7.333	0.046*^a^	3.354	1.206-9.329	0.020*^a^
LVI	1.660	0.696-3.962	0.253	-	-	-
PNI	2.853	1.137-7.154	0.025*^a^	2.590	1.014-6.616	0.047*^a^
Rec. Tumor	1.730	0.519-5.761	0.372	-	-	-
Locoreginal failure (LRF)						
HPVOPC	0.208	0.050-0.868	0.031*^a^	0.210	0.049-0.894	0.035*^a^
≥T3	1.470	0.742-2.912	0.270	-	-	-
ECE vs N+	2.855	1.167-6.985	0.022*^a^	2.939	1.155-7.473	0.024*^a^
N+ vs N0	0.398	0.165-0.960	0.040*^a^	0.468	0.167-1.312	0.149
pos. margins	1.610	0.781-3.317	0.197	-	-	-
LVI	2.063	0.984-4.323	0.055	2.214	0.967-5.067	0.060
PNI	1.844	0.779-4.363	0.164	-	-	-
Rec. Tumor	1.309	0.399-4.289	0.657	-	-	-
Distant failure (DF)						
HPVOPC	0.267	0.082-0.870	0.028*^a^	0.153	0.036-0.644	0.010*^a^
≥T3	1.573	0.838-2.850	0.158	-	-	-
ECE vs N+	3.408	1.621-7.164	0.001*^a^	3.109	1.369-7.035	0.007*^a^
N+ vs N0	0.812	0.336-1.957	0.642	0.759	0.287-2.004	0.578
pos. margins	0.774	0.411-1.458	0.428	-	-	-
LVI	1.947	0.995-9.810	0.052	1.963	0.932-4.137	0.076
PNI	1.463	0.634-3.372	0.372	-	-	-
Rec. Tumor	0.319	0.044-2.324	0.259	-	-	-
Any failure (AF)						
HPVOPC	0.232	0.084-0.643	0.005*^a^	0.161	0.050-0.523	0.002*^a^
≥T3	1.629	0.973-2.728	0.064	1.476^d^	0.822-2.648	0.192
ECE vs N+	3.126	1.668-5.858	<0.001*^a^	3.113	1.566-6.187	0.001*^a^
N+ vs N0	0.604	0.305-1.195	0.148*^a^	0.669	0.302-1.484	0.323
pos. margins	1.018	0.603-1.719	0.947	-	-	-
LVI	1.943	1.107-3.412	0.021*^a^	1.996	1.071-3.720	0.030*^a^
PNI	1.609	0.820-3.155	0.166	-	-	-
Rec. Tumor	0.949	0.344-2.622	0.920	-	-	-
Overall survival (OS)						
HPVOPC	0.277	0.134-0.573	0.001*^a^	0.254	0.116-0.557	0.001*^a^
≥T3	1.385	0.926-2.072	0.113	-	-	-
ECE vs N+	1.684	1.065-2.663	0.027*^a^	1.393	0.834-2.327	0.205
N+ vs N0	1.127	0.675-1.883	0.647	1.984	1.091-3.610	0.025*^a^
pos. margins	0.745	0.497-1.116	0.154	-	-	-
LVI	1.284	0.799-2.064	0.302	-	-	-
PNI	1.666	0.995-2.790	0.052	1.462^d^	0.853-2.506	0.167
Rec. Tumor	0.816	0.357-1.864	0.630	-	-	-
Disease specific survival (DSS)						
HPVOPC	0.076	0.010-0.557	0.011*^a^	0.079	0.011-0.576	0.012*^a^
≥T3	1.839	1.003-3.373	0.049*^a^	1.719	0.910-3.250	0.095
ECE vs N+	2.837	1.366-5.892	0.005*^a^	2.160	1.021-4.571	0.044*^a^
N+ s N0	0.668	0.300-1.486	0.322	1.093	0.484-2.463	0.831
pos. margins	0.522	0.220-1.239	0.140	-	-	-
LVI	1.628	0.832-3.187	0.155	-	-	-
PNI	1.791	0.842-3.812	0.130	-	-	-
Rec. Tumor	0.965	0.298-3.124	0.953	-	-	-
Disease free survival (DFS)						
HPVOPC	0.300	0.156-0.575	<0.001*^a^	0.294	0.146-0.590	0.001*^a^
≥T3	1.463	1.005-2.130	0.047*^a^	1.369^d^	0.873-2.146	0.171
ECE vs N+	2.003	1.305-3.077	0.001*^a^	1.876	1.159-3.035	0.010*^a^
N+ vs N0	1.001	0.618-1.621	0.997	1.684	0.949-2.985	0.075
pos. margins	1.191	0.778-1.824	0.420	-	-	-
LVI	1.512	0.985-2.322	0.059	1.170^d^	0.718-1.906	0.528
PNI	1.604	0.984-2.613	0.058	1.236^d^	0.733-2.081	0.427
Rec. Tumor	0.854	0.397-1.836	0.685	-	-	-

^a^P-values < 0.05 were marked with asterisk

^b^Nodal positive patients with ECE have been compared against nodal positive patients without ECE (ECE vs N+) and nodal positive patients without ECE against patients without nodal disease (N+ vs N0) to calculate the excess risk of ECE to nodal disease only

^c^Close margin vs R0 was neither significant in univariate (HR = 2.200, p = 0.128), nor in multivariate (HR 1.113, p = 0.865) Factors eliminated during the backward-selection are shown for the sake of completeness in grey letters in multivariate analysis

^d^Factors eliminated during the backward-selection are shown for the sake of completeness in multivariate analysis

### HPV- p16 positive oropharyngeal carcinoma

**Fig. 3:**
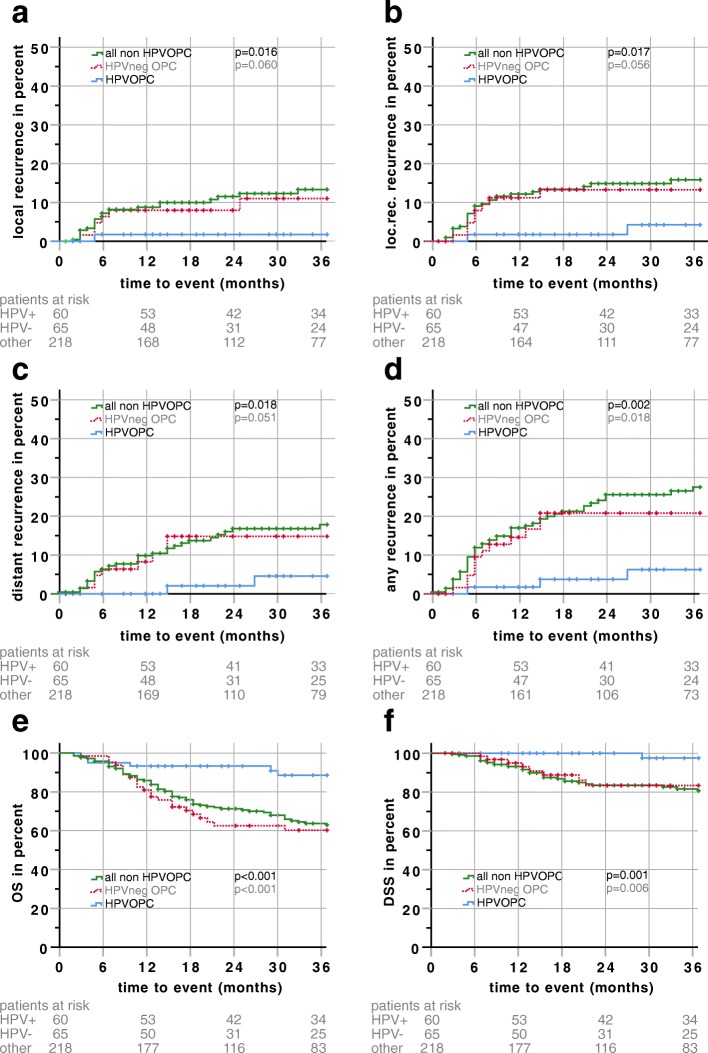
Kaplan-Meier plots for patients with HPV-p16-positive oropharyngeal cancer (HPVOPC, blue line) vs all other Patients (non HPVOPC, green line) HPV-p16 negative oropharyngeal cancer (HPVneg OPC, red dotted line) are shown as reference for oropharyngeal cancer. **a** local recurrence **b** locoregional recurrence (loc.reg.) **c** distant recurrence **d** any recurrence **e** overall survival (OS) and **f** disease-specific survival (DSS); the plot for disease-free survival (DFS) is not shown (*p* < 0.001). Follow-up time was clipped at 36 months. Patients at risk are displayed under the respective plots. Censors are represented by crosses

Patients with HPV-p16 positive oropharyngeal carcinoma (HPVOPC) have been analyzed separately (*n* = 60, estimated median follow-up 45 months). With only one local, one regional and three distant recurrences in 4 patients, the 3-year local, locoregional, distant and overall failure estimates (1.8, 4.2, 4.6 and 6.2%) were significantly lower in patients with HPVOPC compared to all other non HPVOPC (13.3, 15.9, 16.8 and 26.5%) (Fig. 3a-d). The 3-year OS, DSS and DFS rates of HPVOPC were significantly better with 88.6, 97.5 and 85.1% compared to 63.7, 81.6 and 56.4% of all other non HPVOPC. Also, when comparing only within the subgroup of OPC (*n* = 65), OS, DFS, DSS and the overall recurrence rate, were significantly better in HPVpos OPC then in HPV neg OPC (3-year estimates: 60.2, 58.5, 83.4 and 20.8%) (Fig. 3e+f).

## Discussion

The present study represents a well-established and thoroughly followed up cohort of 302 “everyday patients”, treated for tumors of the oral cavity, oropharynx, hypopharynx and larynx with postoperative (chemo) radiation in our clinic between 06/2008 and 08/2015.

A clear limitation of our study is the obvious selection bias by only including irradiated patients and the partly retrospective nature of the study. Additionally, we report a heterogeneous cohort with patients partly treated for recurrence (7.9% or second primaries (2.0%), therefore the results should be interpreted with some caution.

However, the results of our study are in good agreement with previously published cohorts such as RTOG-9501 [17], RTOG-0234 [18], EORTC 22931 [2] and ARO 96–3 [9].

ARO 96–3 is a trial that studied the addition of CDDP and 5-FU to adjuvant radiation in high-risk patients. In ARO 96–3 (chemotherapy arm, 226 patients) and LMU-ARO (ARO-analogue subgroup, 138 patients) 5-year locoregional failure rates were 11.4 and 10.9%, the 5-year distant failure rates were 25.5 and 23% and the 5-year OS rates were 58.1 and 60.6%, respectively.

The patients’ characteristics of our subgroup represents a high-risk group with UICC stage III 16.7%, stage IV 80.7, 32.8% R1, 32.1% close margin and 43.1% ECE, 33.9% LVI. 25.2% of the patients had HPVOPC. Overall, the ARO 96–3 chemotherapy regimen was well tolerated which is indicated by the fact that 87% of the patients completed both cycles chemotherapy as intended. With 138 patients treated, the LMU-ARO subgroup is to our knowledge the largest published cohort of patients treated with CDDP/5-FU, supporting its feasibility and corroborating the preliminary results of the ARO 96–3 study. It should be mentioned, that this regimen was discontinued in 2016 due to the lack of solid published long-term data, adopting CDDP mono as new standard of care.

Also, the results are in line with other studies with PORT-C (EORTC 22931, RTOG-9501, RTOG-0234). Comparing the LMU-ARO subgroup to RTOG-9501 (5-year locoregional failure rate 10.9% vs 22%, 3-year OS 71% vs 56%), the RTOG cohort (PORT 60/2 Gy with CDDP 100 mg/m^2^ BSA) had younger patients with similar rates of positive margins, a comparable proportion of oropharyngeal carcinoma (48% vs 55%) but higher rates of at least two affected lymph nodes and/or ECE (83% vs 67%). In comparison of LMU-ARO to EORTC 22931 (5-year locoregional failure rate 10.9% vs 18%, 5-year OS 60.6% vs 53%), the EORTC (PORT 66/2 Gy with CDDP 100 mg/m^2^ BSA) cohort had also younger patients, comparable rates of positive margins and positive lymph nodes, but higher rates of ECE (61% vs 43%), more T4-Tumors (43% vs 17%) and less oropharyngeal carcinomas (32% vs 55%). The newer RTOG-0234 study compared CDDP+Cetuximab vs Docetaxel+Cetuximab with the docetaxel arm performing better in terms of OS, DFS and distant failure rate. Compared to the RTOG-0234 docetaxel arm (19.9%) and the CDDP arm (19.8%), with 9.7% less locoregional failures after two years were observed in the LMU-ARO subgroup. With a 2-year distant metastasis rate of 16.5%, the LMU-ARO subgroup ranges between the RTOG-0234 docetaxel (13.2%) and the CDDP-Arm (25.0%). The 2-year DFS and OS or the LMU-ARO subgroup was comparable to the Docetaxel-Arm of RTOG 0234 with 69.1% vs 65.9 and 78.1% vs 79.2%, respectively. RTOG 0234 in comparison to the LMU-ARO subgroup had younger patients, more stage IV patients (94% vs 81%), more positive margins (40% vs 33%), more ECE (60% vs 43%), less oropharyngeal carcinoma (34% vs 55%), but the same rate of HPVOPC (26% vs 25,2%).

Twenty-four patients in our cohort have been treated due to recurrence of a previously only surgically treated HNSCC. Interestingly, the recurrence or survival endpoints did not differ significantly from the entire cohort in uni- and multivariate analysis. This could be attributable to a selection bias of only re-operating patients with surgically manageable recurrences (R1-Rate 33.3%), excluding patients with fulminant recurrences and thus selecting for less aggressive tumor biology.

In the recent decade, it became evident that HPVOPC represents a distinct subgroup of patients with a different prognosis [12, 19]. Also, in our cohort, oropharyngeal carcinoma with positive p16 IHC-staining (HPVOPC) performed significantly better for locoregional failure, distant failure, any failure, DSS, DFS and OS (Table 2, Fig. 3). The 2-year locoregional failure, DFS and OS estimates were 1.8, 89.8 and 93.3%. Furthermore, in multivariate analysis, patients with HPVOPC performed significantly better with regards to all endpoints, except for local failure (*p* = 0.064) compared with non HPVOPC. In comparison to other cohorts that analyzed HPVOPC, the failure and survival rates are quite similar (DKTK-ROG [14, 20] p16 IHC: 2-year locoregional failure rage 1.6%, 2-year OS 96.9%, RTOG-0234 p16 subgroup: 2-year DFS 76.2% (Doc), 86.4% (Cis), 2-year OS 100% (Doc), 90.9% (Cis); Vienna [15]: 2-year OS 91%; Japan 5-yr OS 84.9% [21] vs LMU 79.9%) With only one local, one regional and three distant recurrence in the HPVOPC group the need for studies investigating treatment de-escalation in selected patients is underlined [22, 23]. Interestingly, re-classifying the HPVOPC group according to the new UICC 8th edition, a massive stage migration can be observed. Whereas in UICC 7th edition 18.3 and 73.3% of the tumors have been classified as stage III and IV, in UICC 8th edition only 2 tumors are stage III and no tumors qualify for stage IV (see Table 1). 70.0 and 26.7% of the HPVOPC are now classified as stage I or II, reflecting the good prognosis for this disease [19, 24, 25]. As of now, HPVOPC is still being treated similar to non HPVOPC due to the lack of clinical evidence. However, de-escalation strategies are being investigated in clinical trials: The PATHOS trial [26], the ECOG trial [27] the DART-HPV-trial [28], the DELPHI trial [29] and the ADEPT-trial [30] will hopefully provide new evidence for the feasibility and safety of dose or chemotherapy modifications for HPVOPC in the adjuvant setting.

With a high availability of pathological factors, such as resection status (97.4%) ECE (99.0%), LI (90.7%), VI (90.7%) and HPV-p16-status (77.2% of all tumors; 84% of oropharyngeal tumors), a long-time follow-up (median 45 months) and well documented events (26 local, 33 locoregional, 39 distant recurrences and 97 deaths) our study provides valid data for extensive statistical analysis. In uni- and multivariate analysis previously described risk factors could be confirmed, although as a clear limitation the inclusion of only patients in need for adjuvant (chemo)radiation harbors an intrinsic selection bias. In addition, the results of the analysis should be interpreted with caution due to its retrospective nature. By focusing on known risk factors in the analyses, the risk of multiple testing is limited. In multivariate analysis risk factors such as positive resection margins (HR 3.354, *p* = 0.020) and PNI (HR 2.590, *p* = 0.047) were significantly associated with higher risk for local recurrence (Table 2). Close margins were not significantly associated with higher risk for local recurrence (multivariate HR = 1.113, *p* = 0.865), however, in our clinic most patients with close margins had concomitant chemotherapy, thus a concealing effect of the added chemotherapy cannot be ruled out. Additionally, in multivariate analysis, ECE was associated with significantly impaired locoregional (HR 2.939, *p* = 0.024), distant (HR 3.109, *p* = 0.007) and overall (HR 3.113, *p* = 0.001) control as well as impaired disease-specific (HR 2.160, *p* = 0.044) and disease-free (HR 1.876, *p* = 0.010) survival rates. This study is well in line with the literature with ECE being a major risk factor for poor outcome [31, 32]. Interestingly, in multivariate analysis, positive nodal disease (HR 1.984, *p* = 0.025) was a predictor for impaired overall survival without significant additional risk by ECE (HR 1.393, *p* = 0.205). In multivariate analysis, LVI was significantly associated with an increased risk for overall relapses (HR 1.996, *p* = 0.030). For LVI, it has to be taken into account, that only 68 patients had LVI reported by the pathologist, but 193 patients had positive nodal disease, thus a sampling error cannot be excluded. Yet, LVI but not nodal positive disease remained statistically significant in multivariate analysis.

All-in-all, our data indicates that further therapeutic improvements for patients with locally advanced HNSCC are still needed. Interestingly, in our cohort more distant than locoregional failures were observed pointing towards the need of innovative systemic therapies such as immunotherapy [33, 34]. On the other hand, the results for HPVOPC were excellent, supporting the efforts to de-escalate treatment regimens in selected patients with HPVOPC. Apart from the HPV-status, pathological features such as R-Status, LVI, PNI, positive nodal disease and ECE provide solid information for the assessment of the risk of recurrence. However, innovative biomarkers [35–40] might further assist to select appropriate patients for possible treatment modification, such as dose-escalation or de-escalation, intensification or de-intensification of chemotherapy or the addition of immunotherapy [41].

## Conclusion

In conclusion, the adjuvant LMU-KKG-cohort represents the “everyday patient” treated in our facility, affirming previously described risk factors and compares in line with historical cohorts, thus laying a sound foundation for further translational research within the framework of a clinical cooperation group.
